# (9*S*,13*R*,14*R*)-7,8-Didehydro-3,4,7-trimeth­oxy-17-methyl­morphinan-6-one

**DOI:** 10.1107/S1600536808040749

**Published:** 2009-03-06

**Authors:** Yu-Feng Li, Yi Qian, Li-He Yin, Ran Lv, Hong-Jun Zhu

**Affiliations:** a Department of Applied Chemistry, College of Science, Nanjing University of Technology, Nanjing 210009, People’s Republic of China

## Abstract

The title compound, C_20_H_25_NO_4_, was synthesized by a Mitsunobu reaction of sinomenine [(9*S*,13*R*,14*R*)-7,8-didehydro-4-hydroxy-3,7-dimethoxy-17-methylmorphinan-6-one] with methanol. The chiral centers were unchanged during the reaction. Intra­molecular C—H⋯O hydrogen bonds result in the formation of six-membered rings.

## Related literature

For the anti-inflammatory, anti­tussive and anti­arrgythmic activities of sinomenine, see: Wang & Li (1965 ▶).
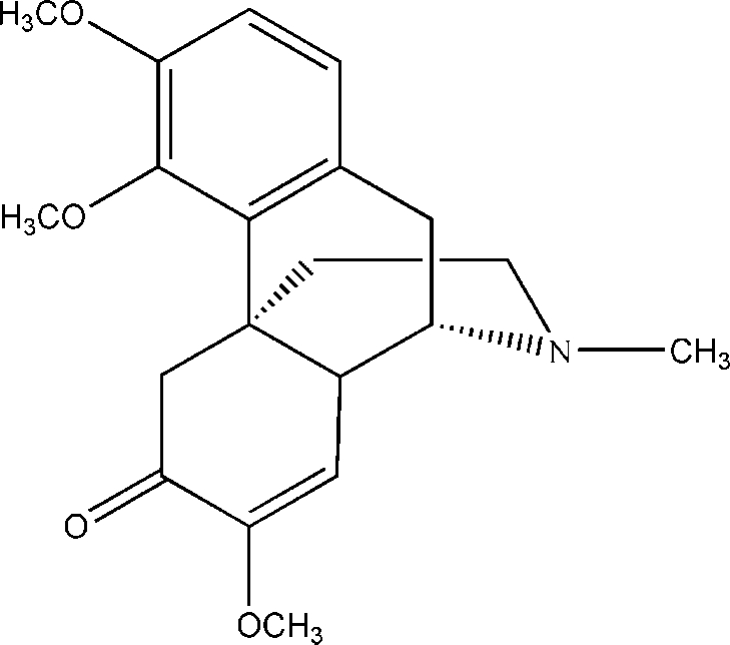


## Experimental

### 

#### Crystal data

C_20_H_25_NO_4_
*M* *_r_* = 343.41
Trigonal, 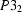

*a* = 10.9590 (15) Å
*c* = 12.726 (3) Å
*V* = 1323.6 (4) Å^3^
*Z* = 3
Mo *K*α radiation
μ = 0.09 mm^−1^
*T* = 298 K
0.40 × 0.30 × 0.30 mm

#### Data collection

Enraf–Nonius CAD-4 diffractometer
Absorption correction: ψ scan (North *et al.*, 1968 ▶) *T*_min_ = 0.965, *T*_max_ = 0.974
2008 measured reflections
1728 independent reflections
1281 reflections with *I* > 2σ(*I*)
*R*_int_ = 0.037
3 standard reflections every 200 reflections intensity decay: none

#### Refinement

*R*[*F* ^2^ > 2σ(*F* ^2^)] = 0.070
*wR*(*F* ^2^) = 0.208
*S* = 1.06
1728 reflections
226 parameters
44 restraints
H-atom parameters constrained
Δρ_max_ = 0.45 e Å^−3^
Δρ_min_ = −0.22 e Å^−3^

### 

Data collection: *CAD-4 Software* (Enraf–Nonius, 1989 ▶); cell refinement: *CAD-4 Software*; data reduction: *XCAD4* (Harms & Wocadlo, 1995 ▶); program(s) used to solve structure: *SHELXS97* (Sheldrick, 2008 ▶); program(s) used to refine structure: *SHELXL97* (Sheldrick, 2008 ▶); molecular graphics: *SHELXTL* (Sheldrick, 2008 ▶); software used to prepare material for publication: *SHELXTL*.

## Supplementary Material

Crystal structure: contains datablocks global, I. DOI: 10.1107/S1600536808040749/hk2561sup1.cif

Structure factors: contains datablocks I. DOI: 10.1107/S1600536808040749/hk2561Isup2.hkl

Additional supplementary materials:  crystallographic information; 3D view; checkCIF report

## Figures and Tables

**Table 1:** Hydrogen-bond geometry (Å, °)

*D*—H⋯*A*	*D*—H	H⋯*A*	*D*⋯*A*	*D*—H⋯*A*
C5—H5*A*⋯O1	0.97	2.23	2.853 (9)	121
C16—H16*B*⋯O1	0.97	2.53	3.102 (10)	118
C18—H18*B*⋯O2	0.96	2.14	2.812 (16)	126

**Fig. 1.**
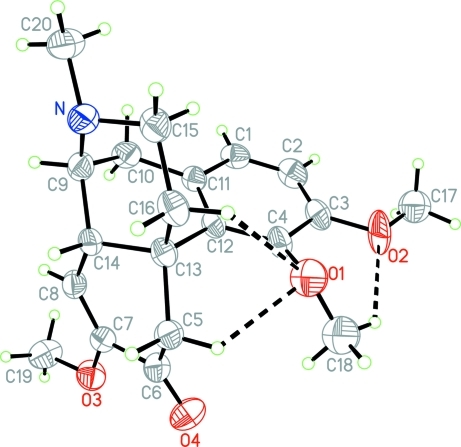
The molecular structure of (I), with the atom-numbering scheme. Displacement ellipsoids are drawn at the 30% probability level. Hydrogen bonds are shown as dashed lines.

## supplementary crystallographic information

### Comment

(9*S*,13*R*,14*R*)-7,8-Didehydro-3,4,7-trimethoxy-17-methylmorphinan-6-one (I) is a derivative of natural sinomenine. Sinomenine was reported possessing anti-inflammatory, antitussive and antiarrgythmic activities (Wang *et al.*, 1965). We report here the crystal structure of I.

The molecular structure of I is shown in Fig. 1. The X-ray diffraction results show that I is a tetracyclic alkaloid with three chiral centers. The piperidine ring is in a chair conformation, and the other two six-membered aliphatic rings are in twisted boat conformation.

The intramolecular C—H···O hydrogen bonds(C5—H5A···O1, C16—H16A···O1 and C18—H18B···O2) result in the formation of six-membered rings, which may be effective to the stabilization of the crystals.

### Experimental

(9*S*,13*R*,14*R*)-7,8-Didehydro-4-hydroxy-3,7-dimethoxy-17-methylmorphinan-6-one (5 mmol, 1.65 g), triphenyl phosphine (10 mmol, 2.62 g) and absolute methanol (1 ml) were added in tetrahydrofuran(THF, 50 ml). Diethyl azodicarboxylate(10 mmol, 1.74 g) was added in dropwise during a period of 30 min. The solution was continuosly stirred for another 12 h. The solution was concentrated under reduced pressure and the residue was purified by column chromatography on silca gel(60–100 mesh) using ethyl acetate/triethylamine (V/V = 10:1). White product was obtained by crystallization from isopropyl ether. Crystals of the product suitable for X-ray diffraction were obtained by slow evaporation of methanol solution.

### Refinement

All H atoms were positioned geometrically, with C—H=0.98, 0.97, 0.96 and 0.93 Å for methine, methylene, methyl and aromatic H atoms,respectively, and constrained to ride on their parent atoms, with *U*_iso_(H)=*xU*_eq_(C), where *x*=1.5 for methyl H atoms and *x*=1.2 for all other H atoms. In the absence of significant anomalous dispersion effects, Friedel pairs were merged by using the instruction of "MERG 3".

### Crystal data

**Table:**

C_20_H_25_NO_4_	*D*_x_ = 1.292 Mg m^−^^3^
*M**_r_* = 343.41	Mo *K*α radiation, λ = 0.71073 Å
Trigonal, *P*3_2_	Cell parameters from 25 reflections
Hall symbol: P 32	θ = 10–13°
*a* = 10.9590 (15) Å	µ = 0.09 mm^−^^1^
*c* = 12.726 (3) Å	*T* = 298 K
*V* = 1323.6 (4) Å^3^	Block, colorless
*Z* = 3	0.40 × 0.30 × 0.30 mm
*F*(000) = 552	

### Data collection

**Table:**

Enraf–Nonius CAD-4 diffractometer	1281 reflections with *I* > 2σ(*I*)
Radiation source: fine-focus sealed tube	*R*_int_ = 0.037
graphite	θ_max_ = 26.0°, θ_min_ = 2.2°
ω/2θ scans	*h* = −13→6
Absorption correction: ψ scan (North *et al.*, 1968)	*k* = 0→11
*T*_min_ = 0.965, *T*_max_ = 0.974	*l* = −15→15
2008 measured reflections	3 standard reflections every 200 reflections
1728 independent reflections	intensity decay: none

### Refinement

**Table:**

Refinement on *F*^2^	Primary atom site location: structure-invariant direct methods
Least-squares matrix: full	Secondary atom site location: difference Fourier map
*R*[*F*^2^ > 2σ(*F*^2^)] = 0.070	Hydrogen site location: inferred from neighbouring sites
*wR*(*F*^2^) = 0.208	H-atom parameters constrained
*S* = 1.06	*w* = 1/[σ^2^(*F*_o_^2^) + (0.1178*P*)^2^ + 0.4849*P*] where *P* = (*F*_o_^2^ + 2*F*_c_^2^)/3
1728 reflections	(Δ/σ)_max_ = 0.005
226 parameters	Δρ_max_ = 0.45 e Å^−^^3^
44 restraints	Δρ_min_ = −0.22 e Å^−^^3^

### Special details

**Table:**

Geometry. All e.s.d.'s (except the e.s.d. in the dihedral angle between two l.s. planes) are estimated using the full covariance matrix. The cell e.s.d.'s are taken into account individually in the estimation of e.s.d.'s in distances, angles and torsion angles; correlations between e.s.d.'s in cell parameters are only used when they are defined by crystal symmetry. An approximate (isotropic) treatment of cell e.s.d.'s is used for estimating e.s.d.'s involving l.s. planes.
Refinement. Refinement of *F*^2^ against ALL reflections. The weighted *R*-factor *wR* and goodness of fit *S* are based on *F*^2^, conventional *R*-factors *R* are based on *F*, with *F* set to zero for negative *F*^2^. The threshold expression of *F*^2^ > σ(*F*^2^) is used only for calculating *R*-factors(gt) *etc*. and is not relevant to the choice of reflections for refinement. *R*-factors based on *F*^2^ are statistically about twice as large as those based on *F*, and *R*- factors based on ALL data will be even larger.

### Fractional atomic coordinates and isotropic or equivalent isotropic displacement parameters (Å^2^)

**Table:**

	*x*	*y*	*z*	*U*_iso_*/*U*_eq_	
O1	0.7890 (7)	0.2303 (6)	0.0383 (5)	0.0855 (17)	
O2	0.8937 (7)	0.0744 (5)	0.1262 (6)	0.093 (2)	
O3	1.2532 (6)	0.7232 (5)	−0.1456 (4)	0.0719 (13)	
O4	1.0260 (6)	0.4750 (6)	−0.1870 (4)	0.0765 (15)	
N	0.9014 (7)	0.6779 (6)	0.2458 (4)	0.0607 (14)	
C18	0.7882 (16)	0.1603 (14)	−0.0423 (8)	0.123 (4)	
H18A	0.6976	0.1188	−0.0757	0.185*	
H18B	0.8071	0.0873	−0.0210	0.185*	
H18C	0.8595	0.2222	−0.0909	0.185*	
C17	0.9251 (11)	−0.0152 (9)	0.1821 (8)	0.093 (3)	
H17A	0.8722	−0.1088	0.1540	0.139*	
H17B	0.9009	−0.0159	0.2547	0.139*	
H17C	1.0240	0.0172	0.1763	0.139*	
C3	0.9567 (7)	0.2137 (7)	0.1507 (5)	0.0592 (16)	
C4	0.9072 (7)	0.2945 (7)	0.0963 (5)	0.0563 (15)	
C12	0.9634 (6)	0.4366 (6)	0.1177 (4)	0.0436 (12)	
C11	1.0594 (6)	0.4957 (7)	0.1981 (4)	0.0470 (13)	
C1	1.1056 (8)	0.4155 (8)	0.2521 (5)	0.0590 (17)	
H1A	1.1723	0.4579	0.3051	0.071*	
C2	1.0563 (8)	0.2771 (8)	0.2294 (6)	0.0653 (19)	
H2A	1.0890	0.2259	0.2663	0.078*	
C19	1.3723 (8)	0.8546 (9)	−0.1173 (7)	0.082 (2)	
H19A	1.4485	0.8759	−0.1646	0.123*	
H19B	1.4005	0.8490	−0.0468	0.123*	
H19C	1.3480	0.9274	−0.1212	0.123*	
C20	0.9242 (11)	0.7233 (10)	0.3554 (6)	0.085 (2)	
H20A	0.9976	0.8201	0.3594	0.127*	
H20B	0.9511	0.6659	0.3949	0.127*	
H20C	0.8388	0.7138	0.3840	0.127*	
C15	0.7923 (7)	0.5297 (7)	0.2337 (5)	0.0569 (16)	
H15A	0.7056	0.5152	0.2655	0.068*	
H15B	0.8213	0.4711	0.2709	0.068*	
C16	0.7647 (6)	0.4843 (7)	0.1193 (5)	0.0531 (15)	
H16A	0.7221	0.5323	0.0842	0.064*	
H16B	0.6994	0.3837	0.1154	0.064*	
C13	0.9034 (6)	0.5194 (6)	0.0638 (5)	0.0461 (13)	
C5	0.8825 (7)	0.4990 (7)	−0.0559 (5)	0.0533 (14)	
H5A	0.8155	0.4009	−0.0706	0.064*	
H5B	0.8429	0.5550	−0.0819	0.064*	
C6	1.0162 (7)	0.5401 (7)	−0.1131 (5)	0.0557 (15)	
C7	1.1414 (7)	0.6762 (7)	−0.0771 (5)	0.0541 (14)	
C8	1.1330 (7)	0.7371 (6)	0.0109 (5)	0.0532 (15)	
H8A	1.2119	0.8200	0.0328	0.064*	
C14	1.0026 (7)	0.6780 (6)	0.0759 (4)	0.0470 (13)	
H14A	0.9502	0.7236	0.0519	0.056*	
C9	1.0334 (7)	0.7134 (7)	0.1920 (5)	0.0549 (15)	
H9A	1.0938	0.8159	0.1966	0.066*	
C10	1.1204 (8)	0.6499 (7)	0.2307 (5)	0.0593 (16)	
H10A	1.1262	0.6562	0.3067	0.071*	
H10B	1.2153	0.7046	0.2031	0.071*	

### Atomic displacement parameters (Å^2^)

**Table:**

	*U*^11^	*U*^22^	*U*^33^	*U*^12^	*U*^13^	*U*^23^
O1	0.090 (4)	0.069 (3)	0.099 (4)	0.040 (3)	−0.017 (3)	−0.012 (3)
O2	0.101 (4)	0.042 (3)	0.140 (5)	0.039 (3)	−0.018 (4)	0.016 (3)
O3	0.071 (3)	0.068 (3)	0.071 (3)	0.030 (3)	0.023 (2)	0.011 (2)
O4	0.077 (3)	0.081 (3)	0.075 (3)	0.042 (3)	−0.001 (3)	−0.024 (3)
N	0.077 (4)	0.066 (3)	0.053 (3)	0.046 (3)	0.007 (3)	0.001 (2)
C18	0.207 (13)	0.138 (9)	0.090 (7)	0.135 (10)	−0.005 (7)	−0.001 (7)
C17	0.112 (7)	0.062 (5)	0.126 (7)	0.060 (5)	0.041 (6)	0.043 (5)
C3	0.059 (4)	0.051 (3)	0.072 (4)	0.031 (3)	0.010 (3)	0.020 (3)
C4	0.058 (4)	0.041 (3)	0.069 (4)	0.024 (3)	0.001 (3)	0.014 (3)
C12	0.048 (3)	0.046 (3)	0.045 (3)	0.030 (3)	0.002 (2)	0.010 (2)
C11	0.056 (3)	0.055 (3)	0.042 (3)	0.037 (3)	−0.001 (3)	0.002 (2)
C1	0.067 (4)	0.085 (5)	0.049 (3)	0.056 (4)	−0.002 (3)	0.009 (3)
C2	0.069 (4)	0.073 (5)	0.077 (4)	0.053 (4)	0.006 (4)	0.021 (4)
C19	0.062 (5)	0.086 (6)	0.078 (5)	0.023 (4)	0.012 (4)	−0.008 (4)
C20	0.124 (7)	0.101 (6)	0.054 (4)	0.074 (6)	0.010 (4)	0.001 (4)
C15	0.061 (4)	0.062 (4)	0.064 (4)	0.042 (3)	0.010 (3)	0.009 (3)
C16	0.045 (3)	0.053 (3)	0.066 (4)	0.028 (3)	0.002 (3)	0.013 (3)
C13	0.047 (3)	0.043 (3)	0.054 (3)	0.027 (3)	−0.004 (3)	0.006 (2)
C5	0.062 (3)	0.046 (3)	0.060 (3)	0.034 (3)	−0.010 (3)	−0.002 (3)
C6	0.064 (4)	0.050 (3)	0.059 (3)	0.032 (3)	−0.002 (3)	0.000 (3)
C7	0.060 (3)	0.054 (3)	0.046 (3)	0.027 (3)	0.004 (3)	0.003 (3)
C8	0.058 (3)	0.045 (3)	0.054 (3)	0.024 (3)	0.003 (3)	0.003 (2)
C14	0.060 (4)	0.042 (3)	0.045 (3)	0.030 (3)	0.004 (3)	0.007 (2)
C9	0.065 (4)	0.052 (3)	0.052 (4)	0.033 (3)	−0.008 (3)	−0.003 (3)
C10	0.069 (4)	0.069 (4)	0.048 (3)	0.041 (4)	−0.019 (3)	−0.008 (3)

### Geometric parameters (Å, °)

**Table:**

O1—C18	1.278 (11)	C19—H19B	0.9600
O1—C4	1.344 (9)	C19—H19C	0.9600
O2—C3	1.360 (8)	C20—H20A	0.9600
O2—C17	1.388 (9)	C20—H20B	0.9600
O3—C7	1.377 (8)	C20—H20C	0.9600
O3—C19	1.424 (9)	C15—C16	1.520 (7)
O4—C6	1.217 (8)	C15—H15A	0.9700
N—C20	1.461 (10)	C15—H15B	0.9700
N—C9	1.466 (9)	C16—C13	1.541 (8)
N—C15	1.466 (9)	C16—H16A	0.9700
C18—H18A	0.9600	C16—H16B	0.9700
C18—H18B	0.9600	C13—C14	1.528 (8)
C18—H18C	0.9600	C13—C5	1.540 (9)
C17—H17A	0.9600	C5—C6	1.490 (10)
C17—H17B	0.9600	C5—H5A	0.9700
C17—H17C	0.9600	C5—H5B	0.9700
C3—C2	1.386 (10)	C6—C7	1.507 (9)
C3—C4	1.428 (8)	C7—C8	1.330 (9)
C4—C12	1.385 (9)	C8—C14	1.491 (9)
C12—C11	1.376 (8)	C8—H8A	0.9300
C12—C13	1.524 (7)	C14—C9	1.523 (8)
C11—C1	1.395 (8)	C14—H14A	0.9800
C11—C10	1.531 (9)	C9—C10	1.516 (9)
C1—C2	1.363 (10)	C9—H9A	0.9800
C1—H1A	0.9300	C10—H10A	0.9700
C2—H2A	0.9300	C10—H10B	0.9700
C19—H19A	0.9600		
			
C18—O1—C4	118.1 (9)	C16—C15—H15A	109.1
C3—O2—C17	120.6 (7)	N—C15—H15B	109.1
C7—O3—C19	114.3 (6)	C16—C15—H15B	109.1
C20—N—C9	112.2 (6)	H15A—C15—H15B	107.9
C20—N—C15	112.5 (6)	C15—C16—C13	110.4 (5)
C9—N—C15	113.3 (5)	C15—C16—H16A	109.6
O1—C18—H18A	109.5	C13—C16—H16A	109.6
O1—C18—H18B	109.5	C15—C16—H16B	109.6
H18A—C18—H18B	109.5	C13—C16—H16B	109.6
O1—C18—H18C	109.5	H16A—C16—H16B	108.1
H18A—C18—H18C	109.5	C12—C13—C14	111.0 (5)
H18B—C18—H18C	109.5	C12—C13—C5	115.9 (5)
O2—C17—H17A	109.5	C14—C13—C5	103.5 (4)
O2—C17—H17B	109.5	C12—C13—C16	108.0 (5)
H17A—C17—H17B	109.5	C14—C13—C16	106.8 (5)
O2—C17—H17C	109.5	C5—C13—C16	111.2 (5)
H17A—C17—H17C	109.5	C6—C5—C13	112.7 (5)
H17B—C17—H17C	109.5	C6—C5—H5A	109.0
O2—C3—C2	124.0 (6)	C13—C5—H5A	109.0
O2—C3—C4	116.6 (6)	C6—C5—H5B	109.0
C2—C3—C4	119.2 (6)	C13—C5—H5B	109.0
O1—C4—C12	117.9 (5)	H5A—C5—H5B	107.8
O1—C4—C3	120.5 (6)	O4—C6—C5	124.2 (6)
C12—C4—C3	120.6 (6)	O4—C6—C7	120.8 (6)
C11—C12—C4	118.8 (5)	C5—C6—C7	115.0 (5)
C11—C12—C13	120.7 (5)	C8—C7—O3	128.1 (6)
C4—C12—C13	119.9 (5)	C8—C7—C6	119.7 (6)
C12—C11—C1	120.2 (6)	O3—C7—C6	112.2 (5)
C12—C11—C10	122.0 (5)	C7—C8—C14	122.8 (6)
C1—C11—C10	117.8 (5)	C7—C8—H8A	118.6
C2—C1—C11	122.1 (6)	C14—C8—H8A	118.6
C2—C1—H1A	119.0	C8—C14—C9	112.7 (5)
C11—C1—H1A	119.0	C8—C14—C13	114.8 (5)
C1—C2—C3	119.1 (6)	C9—C14—C13	109.2 (5)
C1—C2—H2A	120.5	C8—C14—H14A	106.5
C3—C2—H2A	120.5	C9—C14—H14A	106.5
O3—C19—H19A	109.5	C13—C14—H14A	106.5
O3—C19—H19B	109.5	N—C9—C10	119.4 (5)
H19A—C19—H19B	109.5	N—C9—C14	108.8 (5)
O3—C19—H19C	109.5	C10—C9—C14	108.0 (5)
H19A—C19—H19C	109.5	N—C9—H9A	106.7
H19B—C19—H19C	109.5	C10—C9—H9A	106.7
N—C20—H20A	109.5	C14—C9—H9A	106.7
N—C20—H20B	109.5	C9—C10—C11	113.0 (5)
H20A—C20—H20B	109.5	C9—C10—H10A	109.0
N—C20—H20C	109.5	C11—C10—H10A	109.0
H20A—C20—H20C	109.5	C9—C10—H10B	109.0
H20B—C20—H20C	109.5	C11—C10—H10B	109.0
N—C15—C16	112.4 (5)	H10A—C10—H10B	107.8
N—C15—H15A	109.1		
			
C17—O2—C3—C2	1.2 (12)	C12—C13—C5—C6	−59.6 (7)
C17—O2—C3—C4	−173.7 (7)	C14—C13—C5—C6	62.2 (6)
C18—O1—C4—C12	127.8 (8)	C16—C13—C5—C6	176.6 (5)
C18—O1—C4—C3	−64.2 (11)	C13—C5—C6—O4	139.7 (7)
O2—C3—C4—O1	11.0 (10)	C13—C5—C6—C7	−43.8 (7)
C2—C3—C4—O1	−164.1 (7)	C19—O3—C7—C8	−1.0 (11)
O2—C3—C4—C12	178.7 (6)	C19—O3—C7—C6	177.5 (6)
C2—C3—C4—C12	3.6 (9)	O4—C6—C7—C8	−172.3 (7)
O1—C4—C12—C11	162.8 (6)	C5—C6—C7—C8	11.0 (8)
C3—C4—C12—C11	−5.2 (9)	O4—C6—C7—O3	9.1 (9)
O1—C4—C12—C13	−8.4 (9)	C5—C6—C7—O3	−167.6 (6)
C3—C4—C12—C13	−176.4 (6)	O3—C7—C8—C14	177.1 (6)
C4—C12—C11—C1	4.2 (9)	C6—C7—C8—C14	−1.3 (9)
C13—C12—C11—C1	175.3 (6)	C7—C8—C14—C9	150.3 (6)
C4—C12—C11—C10	−176.7 (6)	C7—C8—C14—C13	24.5 (9)
C13—C12—C11—C10	−5.6 (9)	C12—C13—C14—C8	73.0 (6)
C12—C11—C1—C2	−1.5 (10)	C5—C13—C14—C8	−52.0 (6)
C10—C11—C1—C2	179.3 (6)	C16—C13—C14—C8	−169.4 (5)
C11—C1—C2—C3	−0.2 (10)	C12—C13—C14—C9	−54.6 (6)
O2—C3—C2—C1	−175.6 (7)	C5—C13—C14—C9	−179.6 (5)
C4—C3—C2—C1	−0.8 (10)	C16—C13—C14—C9	62.9 (6)
C20—N—C15—C16	177.7 (6)	C20—N—C9—C10	62.0 (8)
C9—N—C15—C16	−53.7 (7)	C15—N—C9—C10	−66.7 (7)
N—C15—C16—C13	53.3 (6)	C20—N—C9—C14	−173.6 (5)
C11—C12—C13—C14	23.7 (7)	C15—N—C9—C14	57.7 (6)
C4—C12—C13—C14	−165.3 (5)	C8—C14—C9—N	168.2 (5)
C11—C12—C13—C5	141.4 (6)	C13—C14—C9—N	−63.0 (6)
C4—C12—C13—C5	−47.6 (8)	C8—C14—C9—C10	−61.0 (7)
C11—C12—C13—C16	−93.2 (6)	C13—C14—C9—C10	67.9 (7)
C4—C12—C13—C16	77.8 (7)	N—C9—C10—C11	76.5 (7)
C15—C16—C13—C12	62.0 (6)	C14—C9—C10—C11	−48.2 (7)
C15—C16—C13—C14	−57.5 (6)	C12—C11—C10—C9	18.3 (9)
C15—C16—C13—C5	−169.8 (5)	C1—C11—C10—C9	−162.5 (6)

### Hydrogen-bond geometry (Å, °)

**Table:**

*D*—H···*A*	*D*—H	H···*A*	*D*···*A*	*D*—H···*A*
C5—H5A···O1	0.97	2.23	2.853 (9)	121
C16—H16B···O1	0.97	2.53	3.102 (10)	118
C18—H18B···O2	0.96	2.14	2.812 (16)	126
